# Pelvic floor rehabilitation in the treatment of women with dyspareunia: a randomized controlled clinical trial

**DOI:** 10.1007/s00192-019-04019-3

**Published:** 2019-07-08

**Authors:** Fariba Ghaderi, Parvin Bastani, Sakineh Hajebrahimi, Mohammad Asghari Jafarabadi, Bary Berghmans

**Affiliations:** 1 Department of Physiotherapy, Faculty of Rehabilitation, Tabriz University of Medical Sciences, Tabriz, Iran; 2 Department of Gynaecology, Woman’s Reproductive Health Research Center, Tabriz University of Medical Sciences, Tabriz, Iran; 3 Tabriz University of Medical Sciences, Tabriz, Iran; 4 Road Traffic Injury Research Center, Tabriz University of Medical Sciences, Tabriz, Iran; 5 Department of Statistics and Epidemiology, Faculty of Health, Tabriz University of Medical Sciences, Tabriz, Iran; 6 Pelvic Care Centre Maastricht, Maastricht University Medical Centre, Maastricht, The Netherlands

**Keywords:** Sexual dysfunction, Dyspareunia, Pelvic floor rehabilitation

## Abstract

**Introduction and hypothesis:**

Dyspareunia, the symptom of painful sexual intercourse, is a common sexual dysfunction in reproductive-aged women. Because of its multifactorial etiology, a multidisciplinary approach may be required to treat it. Musculoskeletal factors play an important role; thus, rehabilitating the pelvic floor and modifying the tone of the pelvic floor muscles (PFMs) may be an effective way to treat this dysfunction. The aim of this randomized controlled clinical study was to evaluate the effects of pelvic floor rehabilitation techniques on dyspareunia.

**Methods:**

Of 84 women, assessed for eligibility, 64 women with dyspareunia were randomized into two groups: the experimental group (*n* = 32) received electrotherapy, manual therapy, and PFM exercises and the control group (*n* = 32) had no treatment while on the waiting list. Evaluations of PFM strength and endurance, sexual function, and pain were made directly before and after 3 months of treatment and at the 3-month follow-up.

**Results:**

Between-group changes showed significant improvement in the experimental group in comparison with control group. Mean difference in the PFM strength (according to the 0-5 Oxford scale) between groups was 2.01 and the mean difference of endurance was 6.26 s. Also, the mean difference in the Female Sexual Function Index score (the score ranges from 2 to 95) was 51.05, and the mean difference in the VAS score was 7.32. All of the changes were statistically significant (*p* < 0.05).

**Conclusions:**

According to the results, pelvic floor rehabilitation is an important part of a multidisciplinary treatment approach to dyspareunia.

## Introduction

Dyspareunia is the complaint of persistent or recurrent pain or discomfort associated with attempted or complete vaginal penetration [1]. According to the Diagnostic and Statistical Manual of Mental Disorders-V-text revision (DSM-V-T), dyspareunia is sub-classified in genito-pelvic pain/penetration disorders in which a woman experiences recurrent genital pain before, during, or after vaginal penetration [1]. In the USA, the reported prevalence of dyspareunia was 8% to 21% of women [2]. However, because of cultural differences, the prevalence of dyspareunia is very different in undeveloped or developing countries. In these countries, most women with dyspareunia experience pain during sexual intercourse in their lifetimes, but they do not report their pain or seek treatment because of shame or other cultural factors such as gender superiority. For example, in a study in Iran of 319 women between the reproductive ages of 15 and 49 years, 54.5% of the women had dyspareunia [3].

Despite the high prevalence of dyspareunia, few clinical trials have been performed on the diagnosis and treatment of dyspareunia. It has been proposed that the possible causes of dyspareunia are multifactorial, including pelvic floor injury during vaginal delivery, pelvic inflammatory disease, infection, interstitial cystitis, adhesions, sexual violence, or sexual abuse [4], in addition to psychosocial factors, such as depression, anxiety, or other psychological disorders [5]. The musculoskeletal elements of the pelvic floor play an important role in dyspareunia. Previous literature showed that the pelvic floor muscles (PFMs) become weak and overactive simultaneously in dyspareunia [6, 7].

Because of its multifactorial etiology, a multidisciplinary approach is required to treat dyspareunia. Pelvic floor rehabilitation is an important part of this multidisciplinary treatment approach [8, 9]. An expert physiotherapist in pelvic floor rehabilitation uses different manual techniques (such as myofascial release, intra-vaginal massage techniques, etc.) and modalities (such as transcutaneous electrical neural stimulation [TENS], functional electrical stimulation [FES], heat and cold) to treat dyspareunia. Trigger and tender points have been reported to be one of the musculoskeletal sources of dyspareunia; thus, pelvic floor physiotherapy including manual techniques can play an important role in treating dyspareunia [10]. Manual techniques increase the woman’s awareness of her PFMs, may release trigger and tender points, normalize the overactivity, and increase the strength of the PFMs [11]. These aims are reported to be achieved by PFM exercises with or without biofeedback, myofascial release techniques, deep intravaginal massages, and electrotherapeutic modalities [11–13].

According to Bo et al. and Fisher, education also plays an important role in the treatment of dyspareunia [14, 15]. Instructing a woman about the anatomy and function of the PFMs and educating her on how to self-control the activity of these muscles are very important parts of the treatment. In this way, she can relax her pelvic floor muscles when she wants and contract them when needed.

Biofeedback to develop or increase awareness of the patient is an important adjunct to supporting a patient how to realize normal activity of the pelvic floor muscles [16]. Biofeedback by digital palpation is an effective way of achieving this goal because in doing so the pelvic physiotherapist instructs and educates the patient to find and feel the pelvic floor muscles as a prelude to forcing/strengthening the pelvic floor; although it takes more time, it may be more effective than instrumental biofeedback [8, 15].

Myofascial release techniques and intravaginal massage can be useful in improving vascularization and releasing muscle trigger points in the pelvic floor and, thus, can be efficient in treating pain and sexual dysfunction [15–17]. Many of the relevant studies on dyspareunia focused only on pain, and did not examine the strength, activity, and tone of the PFMs.

According to previous studies, pelvic floor rehabilitation is an effective approach in the treatment of dyspareunia [10–17]. However, most of these studies were retrospective or observational. The aim of the present study was to evaluate the effects of pelvic floor rehabilitation on dyspareunia using a randomized controlled clinical trial.

## Materials and methods

A randomized controlled clinical trial (RCT) was performed to evaluate the effects of pelvic floor rehabilitation on adult women with dyspareunia. The study was approved by the Ethics Committee of Tabriz University of Medical Sciences (9349/2014) and was performed at the Department of Physiotherapy. This trial was registered in the Iranian Registry of Clinical Trials (IRCT 2014070118311 N1). All of the participants who attended the study signed a standard written consent form. The study design was controlled by the CONSORT checklist for RCTs.

Eighty-four women with dyspareunia were assessed for eligibility.

The clinical trial involved 64 women with dyspareunia who were referred to the physiotherapy clinic by our urogynecologist. The urogynecologist did all of the necessary examination and relevant laboratory tests to exclude any other causes of dyspareunia except for muscular problems.

The sample size of the study was calculated using Pocock’s formula based on the main outcomes of the study Female Sexual Function Index (FSFI). Considering a confidence interval of 0.95, a power of 0.8 using information obtained from the study pilot, at least 29 patients were needed per group and this was increased to 32 patients per group taking into account a dropout rate of 10%.

**Fig. 1:**
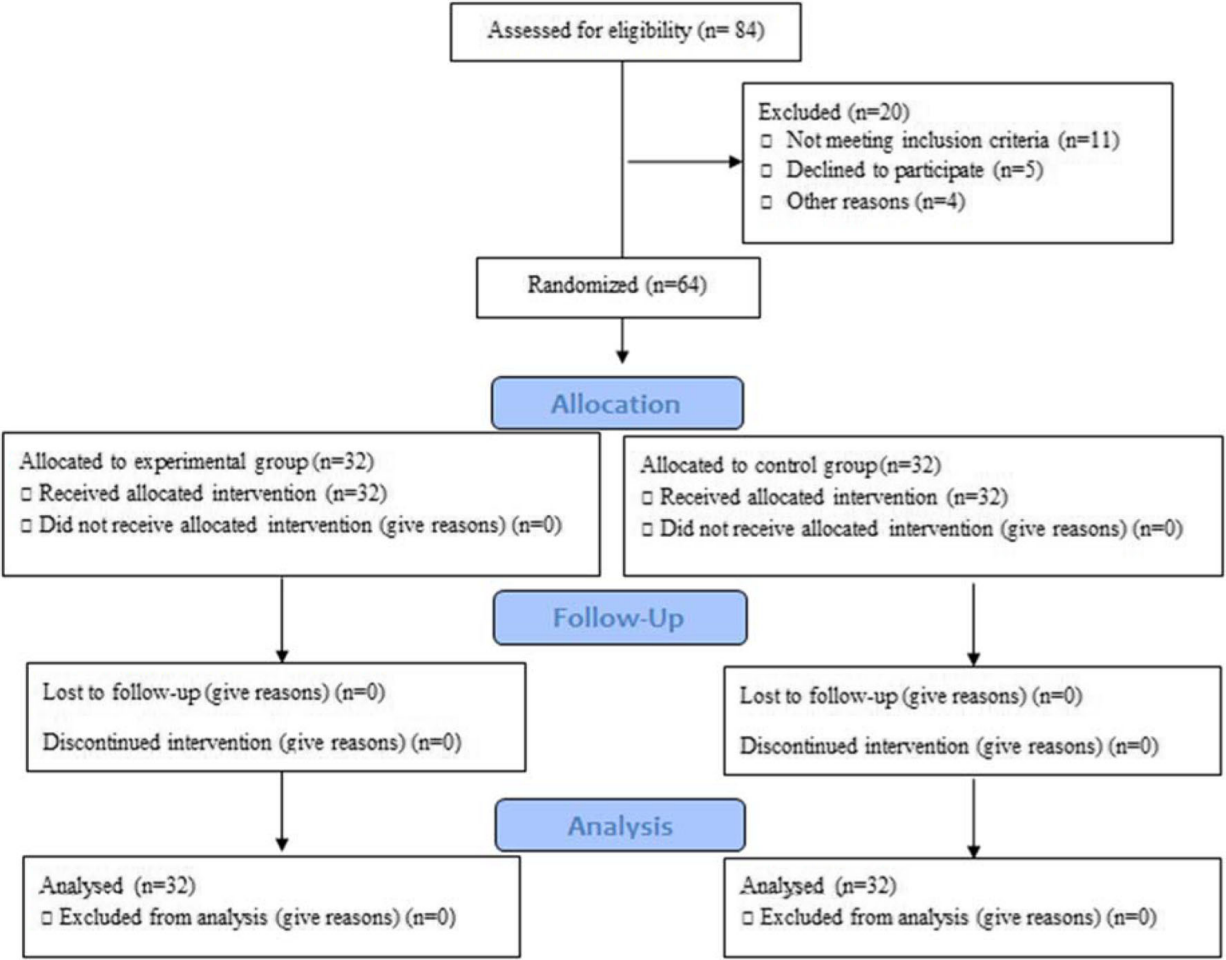
CONSORT 2010 flow diagram of the study

Participants in this trial met the following inclusion criteria: pain in the genital area before, during, or after vaginal intercourse, and pain was greater than 8 on a 10-cm visual analog scale (VAS). If the women had any history of other pathophysiological conditions, such as infections, tumors, major psychiatric illnesses, vaginism, vestibulodynia, vulvar dermatological conditions, painful bladder syndrome or interstitial cystitis, endometriosis, pregnancy, surgery on pelvic organs, or any ongoing treatment for dyspareunia, they were excluded from the study. The flow diagram is shown in Fig. 1.

The participants were randomized using random allocation software (RAS) into two groups; a randomized block procedure of size 2 was used [18]. Concealment of group allocation was performed.

Blinding was only in place for the assessor and the statistician. Because of the nature of the intervention and control, participants and physiotherapists could not be blinded.

The experimental group received physiotherapy treatments once a week for 3 months, whereas the control group was put on a waiting list and received no treatment. It was preferred not to give them a routine stretching or strengthening exercise program because it could influence the PFMs and confound any results. The state clinic had a long waiting list and if a patient did not want to be added to the waiting list, she was referred to another clinic.

A specialized physiotherapist in pelvic floor rehabilitation assessed the participants without knowing group allocations. A standardized and structured functional vaginal examination was performed by digital palpation to examine pelvic floor contractions and relaxation, whereas strength and endurance of the PFMs were evaluated using the modified Oxford scale (0-5 grade scale) [9] The severity of pain at tender or trigger points of the pelvic floor was evaluated using a VAS. The VAS is a 10-cm line on which the patient exactly indicates the severity of her pain (0 = no pain; 10 = extreme pain) [19].

A standardized Persian version of the FSFI [20] was also used in this study. This questionnaire consists of six parts that evaluate desire (2–10 score), arousal (0–20 score), lubrication (0–20 score), orgasm (0–15 score), satisfaction (0–15), and painless intercourse (0–15 score); the total score ranges from 2 to 95. All of the outcomes, including the pelvic floor strength and endurance, and VAS and FSFI scores, were measured directly before the start of the study, directly after the end of the study, i.e., after 3 months of treatment, and at the 3-month follow-up.

In the experimental group, another specialized physiotherapist explained the anatomy and function of the PFMs to each patient at the first session using simple and understandable words. During the vaginal examination, feedback of the PFM activity by digital palpation was given to the patient. The vaginal examination took 20 min or more during the first session. In this way, realizing the increase in the patient’s awareness of the PFM activity increased, she was prepared to better use and control the activity of her PFMs.

Participants in the experimental group received 10 sessions of treatment (once a week) for 3 months and they did progressive pelvic floor muscle exercises at home every day. They did not receive any treatment during menstruation. Each session contained 15–20 min of manual techniques to release trigger points in the pelvic floor using intravaginal myofascial soft-tissue release and deep intravaginal massage, and 20–25 min of high frequency TENS using intravaginal electrodes (at 110 Hz for an 80-ms pulse duration and maximal tolerable intensity to relieve pain). The participants were also instructed to perform graded pelvic floor muscle exercises (PFMEs) and were given instructions on how to perform these exercises progressively each week.

A written instruction and an educational video CD for home exercises and a diary checklist for controlling their daily exercise was given to them to increase their adherence to the PFME.

Statistical analysis was performed using SPSS software (version17; SPSS, Chicago, IL, USA). Normality of the numeric variables was checked and confirmed using the Kolmogorov–Smirnov test. Data were presented using mean (SD), median (min–max) for the numeric normal and non-normal variables respectively and frequency (percentage) for categorical variables. The between-group comparisons of baseline measures and demographic variables were carried out using the independent Student’s *t* test, and/or Chi-squared test where appropriate. For within-group before and after intervention comparisons, repeated measures ANOVA were used. To assess the effect of intervention, the analysis of covariance (ANCOVA) was used to control for baseline measures and confounders. In all analyses, *p* values <0.05 were considered statistically significant.

## Results

Sixty-four out of 84 eligible women were included and randomized to the experimental (*n* = 32) or to the control group (*n* = 32).

At the beginning of the study, the two groups had similar baseline characteristics, with the exception of the severity of pain and the PFM strength and endurance. Table 1 shows the baseline characteristics of the participants in the two groups.

**Table 1:** Baseline characteristics of study participants

Variables	Experimental group (*n* = 32)	Control group (*n* = 32)	*p* value
Age	34.94 (9.15)	35.72 (8.01)	0.718
Height	158.13 (8.87)	159.81 (9.54)	0.467
Weight	70.34 (11.49)	69.09 (12.50)	0.679
BMI	28.13 (3.99)	26.97 (3.98)	0.247
VAS	9.03 (0.86)	8.34 (0.97)	0.004*
FSFI	31.16 (8.31)	35.25 (10.04)	0.081
PFM strength	1.72 (0.72)	2.50 (0.88)	0.000*
PFM endurance	4.53 (2.30)	6.44 (2.78)	0.004*

Data are presented as mean (SD)

*BMI* body mass index, *VAS* visual analog scale, *FSFI* Female Sexual Function Index, *PFM* pelvic floor muscle

*p < 0.05, unpaired Student *t* test

Comparison of the two groups showed that there were significant differences between groups after treatment (Table 2).

**Table 2:** Analytical statistics before/after results between two groups

Variable		Experimental group (*n* = 32)	Control group (*n* = 32)	MD (95% CI) between groups
Desire	Before	3.59 (1.60)	3.56 (1.52)	0.031 (−0.813 to 0.750)
	After	8.38 (1.28)	4.00 (1.52)	4.409 (3.697 to 5.121)**
Arousal	Before	5.06 (2.35)	4.56 (2.04)	0.500 (−1.602 to 0.602)
	After	14.47 (1.86)	5.03 (1.30)	9.310 (8.495 to 10.125)**
Lubrication	Before	4.44 (1.93)	5.25 (2.92)	0.813 (−0.428 to 2.053)
	After	14.44 (1.77)	5.94 (3.61)	9.026 (7.758 to 10.294)**
Orgasm	Before	3.63 (1.89)	4.13 (1.80)	0.500 (−0.426 to 1.426)
	After	11.56 (1.39)	4.91 (1.59)	6.709 (5.924 to 7.495)**
Satisfaction	Before	4.31 (1.37)	4.53 (1.79)	0.219 (−.581 to 1.019)
	After	10.53 (1.10)	4.97 (2.07)	5.666 (4.882 to 6.451)**
Painless	Before	3.88 (1.94)	4.28 (1.87)	0.406 (−0.548 to 1.360)
	After	12.78 (1.38)	4.81 (1.58)	8.073 (7.260 to 8.886)**
FSFI	Before	31.16 (8.31)	35.25 (10.04)	4.094 (−0.514 to 8.701)
	After	88.59 (4.92)	38.69 (6.72)	51.051 (48.274 to 53.828)**
PFM strength	Before	1.72 (0.72)	2.50 (0.88)	0.781 (0.378 to 1.185)**
	After	4.19 (0.72)	2.47 (0.80)	2.014 (1.641 to 2.378)**
PFM endurance	Before	4.53 (2.30)	6.44 (2.78)	1.906 (0.631 to 3.182)**
	After	12.25 (2.07)	6.56 (2.59)	6.267 (5.084 to 7.450)**
VAS	Before	9.03 (0.86)	8.34 (0.97)	−0.68 (−1.14 to −0.22)
	After	1.66 (1.09)	8.72 (1.14)	7.32 (6.76 to 7.88)**
	After 3 months	1.41 (1.10)	8.87 (0.83)	7.57 (7.03 to 8.10)**
	*p* value for RMANOVA	<0.001*	0.059*	

*CI* confidence interval, *MD* mean difference, *RMANOVA* repeated measures ANOVA

All values are presented as mean (SD)

*RMANOVA

**p < 0.05, between group analyses of covariance adjusted for baseline measurements

According to the results, between-group changes showed significant improvement in the experimental group in comparison with the control group. Mean difference in the PFM strength (according to the 0-5 Oxford scale) between groups was 2.01 and the mean difference of endurance was 6.26 s. Also, the mean difference in the FSFI score (the score ranges from 2 to 95) was 51.05. All of the changes were statistically significant (*p* < 0.05).

**Fig. 2:**
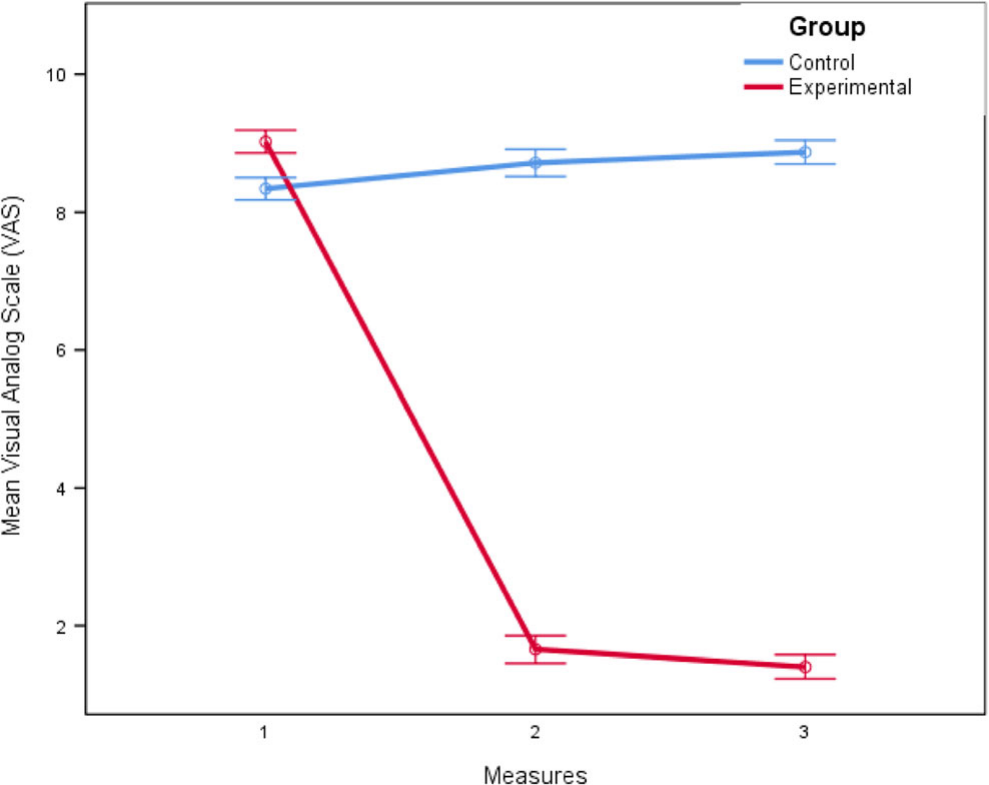
The trend of visual analog scale (VAS) changes before/after and follow-up measurements in both groups

The VAS changes decreased dramatically in the experimental group during the study, and the mean difference in the VAS score before and after treatment was 7.32. Three months after the last treatment session, the superiority of the experimental to control group in VAS continued (mean difference between groups was 7.57; Fig. 2).

## Discussion

This study showed that our pelvic floor rehabilitation program improved genito-pelvic pain, sexual function, PFM strength, and endurance in women with symptomatic dyspareunia. Our pelvic floor rehabilitation program consisted of digital biofeedback, intravaginal manual techniques, supervised PFMEs, and electrotherapy.

According to a recent systematic review (2014) on the role of biofeedback and soft-tissue mobilization in the treatment of dyspareunia, only four relevant studies could be identified in patients with dyspareunia [16]. Each of these studies had a different design and methodology. The studies used biofeedback and soft-tissue release with or without PFME; overall, the results showed significant improvement in pain and sexual dysfunction with subjective measurements [12, 21–23].

Two recent clinical trials have shown similar results by treating dyspareunia patients with PFME and myofascial release [24, 25].

The new clinical guideline for vulvodynia including overactive pelvic floor suggested manual therapy techniques, pelvic floor reeducation treatments, behavioral and lifestyle modification, and PFME for treatment of dyspareunia [26].

In general, our study cannot be compared with previous studies because of the differences in treatment protocols. Most studies used instrumental biofeedback, whereas digital biofeedback by vaginal palpation was used in ours, as recommended by Fisher in 2007 [15], who showed that this type of biofeedback may be more effective than instrumental biofeedback.

Digital biofeedback is a simple but effective way of enhancing a patient’s awareness of her PFMs [15]. Such an intervention does not need any device, but is highly dependent on the practical skills, knowledge, and experience of a highly educated pelvic physiotherapist, together with perseverance, time, and patience.

Digital biofeedback has also been reported to be effective in teaching a patient to control the activity of PFMs especially during intercourse [15]. In our study, we have shown that in the experimental group, it did indeed serve to significantly restore painless intercourse by breaking the vicious pain–spasm cycle.

To our knowledge, our study showed for the first time, comparing an experimental group with a control group without treatment, that in women with musculoskeletally and myofascially based dyspareunia intravaginal manual techniques, including massage and myofascial release of PFMs, result in significantly relaxing PFMs, diminishing over-activity of the PFMs, and a significant decrease in genito-pelvic pain during sessions.

Because other causes of dyspareunia such as infections, endometriosis, etc., were excluded, we tried to investigate a more homogeneous patient population with musculoskeletally and myofascially based genito-pelvic pain. Although a recent review reported doubts about the existence of myofascial trigger points, our digital techniques focused on the treatment of myofascial trigger points and the relaxation of PFMs enabling significant pain reduction [27].

As dyspareunia is related to a decrease in the strength and endurance of PFMs, in addition to overactivity [9], PFMEs were added to the treatment protocol. We decided to incorporate a supervised PFME program because of the apparent [8, 28, 29] superiority of supervised to non-supervised PFME.

Our patients in the experimental group received 12 weeks’ treatment aiming to improve their pelvic floor muscle strength and endurance. Next to this intensive supervised exercise program once a week we sought to control the patient’s daily home exercises by using a diary checklist to support compliance and adherence. This strategy of combined clinic and at home-based PFME program has been reported to be effective for urinary incontinence [28, 29], but has not been investigated so far in patients with dyspareunia.

As Naess and Bø have shown that maximal voluntary pelvic floor muscle contraction can reduce vaginal resting pressure and resting electromyography activity [30], it can be concluded that using maximal contractions may help in overactive pelvic floor to reduce tone.

After 3 months of the pelvic floor rehabilitation under investigation, there was a significant improvement in PFM strength and endurance. In a study by Murina et al., PFMEs were one part of the treatment protocol, but unfortunately the authors did not report any data on PFM strength or endurance [21]. Because of this, their study cannot be compared with the present study.

Another strategy for reducing genito-pelvic pain is transcutaneous electrical nerve stimulation (TENS). TENS may inhibit pain based on the so-called gate control theory [31]. In women in whom digital intravaginal techniques increased pain in particular, we first used TENS to reduce genito-pelvic pain.

In our study, we measured sexual function using the FSFI questionnaire. The results of the FSFI questionnaire showed that all domains, i.e., desire, arousal, lubrication, orgasm, satisfaction, and painless intercourse, were significantly improved after treatment. Because dyspareunia affects a woman’s quality of life and marital relationship, subjective improvement of sexual function plays an important role in preserving her marriage and family life, especially in a patriarchal society like Iran.

Women with dyspareunia feel pain during intercourse, which is induced by trigger points in the PFMs. Relevant literature indicates that transvaginal techniques, such as myofascial release techniques and massage, improve the circulation in the PFMs and release taut bands of muscles and trigger points to break the cycle of genito-pelvic pain and PFM over-activity [17, 26]. Digital biofeedback and education of the patient as elements of the multifaceted intervention also play important roles in alleviating sexual pain and PFM overactivity. Our follow-up measurements showed that women in the experimental group continued to experience the effects of treatment 3 months later.

It was our hypothesis that genito-pelvic pain and sexual dysfunction during intercourse should be treated with a multifaceted intervention consisting not only of massage and release techniques, but also PFM training to improve both PFM strength and endurance. Our results showing significant improvement using the FSFI as a valid subjective outcome and the modified Oxford scale and VAS as objective primary outcome measures support this hypothesis.

Another recent systematic review (2018) was carried out to investigate physiotherapy effects in treating sexual pain disorders in women. In line with our study results, in that systematic review, the conclusions were that increasing muscle awareness and proprioception, improving muscle relaxation, restoring normal activity of PFMs, and increasing the elasticity of the tissues can help to reduce pain in sexual pain disorders [10].

The effect of PFME on pre- and postnatal female sexual function have been reviewed systematically and showed that PFME alone improved postnatal sexual function [32].

Therefore, it can be concluded that the complexity of the diagnosis and treatment of dyspareunia requires a dedicated multidisciplinary team to share their findings so as to understand the causes of dyspareunia [33]. Physical therapists may therefore greatly contribute to treating dyspareunia signs and symptoms. After a comprehensive assessment, multimodal treatment, including biofeedback, manual techniques, electrotherapy, and PFMEs could be useful tools in physiotherapists’ hands to manage the pain and symptoms of dyspareunia. Physiotherapists may provide simple anatomical information from the pelvic floor and teach the patient how to control her pelvic floor tone using a mirror, digital vaginal palpation, or biofeedback. If the physiotherapist finds that there are tender or trigger points in the pelvic floor, minimally invasive methods such as myofascial release and pain relief electrotherapy methods such as TENS can help with the management of pain.

Most dyspareunia patients have overactive PFMs and at the same time weak muscles; thus, PFMEs can help to strengthen the pelvic floor and at the same time reduce pelvic floor resting tone. To participate in the multidisciplinary team to assess and treat dyspareunia adequately, a highly educated and well-trained pelvic physiotherapist is needed.

One of the most important limitations of this study was the absence of objective outcomes, such as EMG or perineometer measurements for PFM strength and endurance.

## Conclusion

According to the results, pelvic floor rehabilitation is an important part of a multidisciplinary treatment approach to dyspareunia.
